# Automatic Labeled Dialogue Generation for Nursing Record Systems

**DOI:** 10.3390/jpm10030062

**Published:** 2020-07-16

**Authors:** Tittaya Mairittha, Nattaya Mairittha, Sozo Inoue

**Affiliations:** Graduate School of Engineering, Kyushu Institute of Technology, 1-1 Sensui-cho, Tobata-ku, Kitakyushu-shi, Fukuoka 804-8550, Japan; fah@sozolab.jp (N.M.); sozo@sozolab.jp (S.Z.)

**Keywords:** nursing record systems, natural language understanding, dialogue systems, machine learning

## Abstract

The integration of digital voice assistants in nursing residences is becoming increasingly important to facilitate nursing productivity with documentation. A key idea behind this system is training natural language understanding (NLU) modules that enable the machine to classify the purpose of the user utterance (intent) and extract pieces of valuable information present in the utterance (entity). One of the main obstacles when creating robust NLU is the lack of sufficient labeled data, which generally relies on human labeling. This process is cost-intensive and time-consuming, particularly in the high-level nursing care domain, which requires abstract knowledge. In this paper, we propose an automatic dialogue labeling framework of NLU tasks, specifically for nursing record systems. First, we apply data augmentation techniques to create a collection of variant sample utterances. The individual evaluation result strongly shows a stratification rate, with regard to both fluency and accuracy in utterances. We also investigate the possibility of applying deep generative models for our augmented dataset. The preliminary character-based model based on long short-term memory (LSTM) obtains an accuracy of 90% and generates various reasonable texts with BLEU scores of 0.76. Secondly, we introduce an idea for intent and entity labeling by using feature embeddings and semantic similarity-based clustering. We also empirically evaluate different embedding methods for learning good representations that are most suitable to use with our data and clustering tasks. Experimental results show that fastText embeddings produce strong performances both for intent labeling and on entity labeling, which achieves an accuracy level of 0.79 and 0.78 f1-scores and 0.67 and 0.61 silhouette scores, respectively.

## 1. Introduction

Task-oriented dialogue systems or virtual assistants are designed to assist users in solving a specific task with explicit intent within minimal dialogue turns. As smart speakers (e.g., Amazon Alexa (https://www.alexa.com), Google Home (https://store.google.com/product/google_home), Apple HomePod (https://www.apple.com/homepod), and Microsoft Cortana (https://www.microsoft.com/en-us/cortana)) become more sophisticated and robust in homes, their utility in clinical settings will grow as well. The most apparent use for voice assistants is transforming electronic health record (EHR) interactions. This field has received significant attention in recent years, not only among academic communities but also in clinical settings [1,2,3].

With the advancement in machine learning, agents can understand the user’s speech in audio signals and convert text into speech. They provide a natural language for the free text of medical records, which contain valuable patient-specific information and a nuanced reflection. They further help to increase sterility and speed up the process by use of the hands-free or voice-activated modes, thus enabling nurses to have more time for direct patient care. Still, several challenges have continued to pose obstacles to the unstructured data when compared to the structured data. The extraction of medical events and their attributes from unstructured clinical notes are challenging to read and categorize with straightforward algorithms. In other words, we can probably still see incomplete responses from these systems and accept that they may not correctly understand human language the way humans perceive it. However, any inaccurate information, such as with symptoms, diagnosis, or medical documents in healthcare can have disastrous outcomes.

Traditional rule-based systems can be circumvented, but are often hand-crafted features which require constant manual intervention for improvement [4,5,6,7,8]. Presently, algorithms that learn from labeled training data are commonly used to handle these problems. As we can see, well-known chatbot platforms (e.g., Amazon Lex (https://aws.amazon.com/lex), Google Dialogflow (https://dialogflow.com), IBM Watson (https://www.ibm.com/watson), and Microsoft LUIS (https://www.luis.ai)) have comprehensive natural language processing (NLP) capabilities. They still need a sufficient number of labeled data to learn a given intent. Most of the previous novel research has also been focused primarily on supervised machine learning algorithms [9,10,11,12,13,14,15]. Existing datasets used to train these models usually rely on human-labeled datasets. One conventional technique is using the Wizard-of-Oz (WOZ) in which trained agents and crowd-sourced workers interact to complete the task [16,17,18,19,20,21]; however, it is labor-intensive and time-consuming. Furthermore, assessing the quality of labels is a difficult problem because it is highly subjective. The problems arise particularly when dealing with domain-specific tasks, such as a nursing domain that requires experts to define knowledge [22,23,24] and consequently involves enormous costs. Accordingly, automated data labeling and processing approaches with little to no human involvement are viable and scalable solutions to handle these matters.

**Figure 1:**
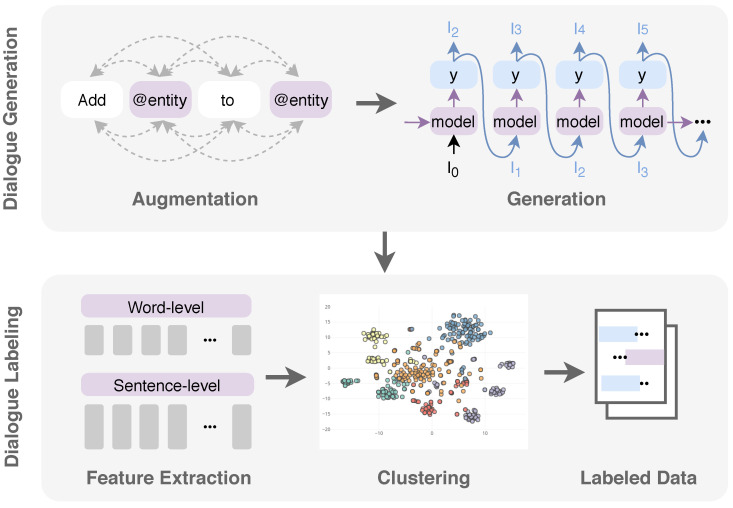
The proposed automatic labeled dialogue generation for nursing record systems, consisting of two tasks—a dialogue generation task and a dialogue labeling task.

In this paper, we propose an automatic dialogue labeling framework that can be used to train Natural Language Understanding (NLU) modules specifically for recording care activities in nursing homes. NLU is considered a core component in implementing task-oriented dialogue systems, which helps to understand the purpose of a user’s utterances (i.e., intent) and extract pieces of valuable information present in the utterance (i.e., entity). As an example, consider an utterance “I have finished preparing a syringe with 2.5 cc of vitamin B12 to a patient’s room 303”, where a dialogue utterance is labeled with intent #prepare-injection, and the following entities are mentioned: @drug-type = vitamin B12; @shot = 2.5 cc; target-resident = patient’s room 303. Figure 1 shows an overview of our proposed method. In essence, our main contributions are the following:

1. **Dialogue Generation:** Due to the lack of an utterance of the nurse transcript (a cold start problem) and expertise from developers in the nursing domain, we develop a data augmentation-based framework to create initial training utterances. The results show that our dataset qualifies 4.71 fluency and 4.66 accuracy scores with crowd-sourced human judgments. This method can be used for other tasks, in which training data is unavailable. Furthermore, we explore scalable ways to generate new statements by computing the character-based model based on long short-term memory (LSTM) [25]. The model achieves an accuracy level of 90% and shows the generated reasonable outputs, which look similar to utterances from the original dataset (0.76 BLEU scores on average).
2. **Dialogue Labeling:** We propose a semantic similarity-based clustering model for intent and entity labeling. To obtain similarity features between utterances, we compare several word-level and sentence-level embedding models to get the best result. The embeddings used are as follows: word-level models include Word2Vec [26], fastText [27], and embeddings from language models (Elmo) [28]; sentence-level include the Universal Sentence Encoder (USE) [29], InferSent [9] Bidirectional Encoder Representations from Transformers (BERT) [30], and Elmo fixed mean-pooling. The evaluation results show that the fastText embeddings model outperforms other embeddings by achieving 0.79 and 0.78 f1-scores and 0.67 and 0.61 silhouette scores of intent and entity clustering.

The paper is organized as follows: First, we describe the background of our study and examine the related works involving the different strategies applied to text augmentation and feature extraction in Section 2. In Section 3, we introduce our proposed dialogue generation method. In Section 4, we explain the implantation of a dialogue labeling task. In Section 5, we present a performance evaluation. In Section 6, we discuss the result obtained. Finally, we conclude the paper with future direction in Section 7.

## 2. Background & Related Work

In this section, we divide the backgrounds into four subsections. First, we explain the basic concept of a task-oriented dialogue system, NLU components, and related works involving entity extraction on EHR data. Second, we describe the challenge of implementing the dialogue labeling framework for the nursing field. Third, we present data augmentation procedures that can be applied in our sequence generation processes. Finally, considering we mainly focus on the process of feature extraction, we provide an overview of previous work in traditional feature engineering methods and more advanced strategies that often leverage machine-learning and deep-learning models, which are involved in our work.

### 2.1. Task-Oriented Dialogue Systems

Dialogue systems are automatic systems that mimic human conversations using text or spoken language [31]. These systems are usually divided into two different groups: First is task-oriented dialogue systems designed for a particular task and set up to have short conversations [32], such as a voice for documentation or health information-seeking for patients; and second are non-task-oriented dialogue systems that are designed for unstructured conversation as a conversation between humans and to maximize long-term user engagement.

NLU is considered a core component in implementing task-oriented dialogue systems, which helps to understand and interpret human language. It consists of two main parts: an intent and an entity. Intents represent the purpose of a user’s utterances, each of which contains different expressions that can be spoken by the user. For example, when talking about how headaches are treated, many different statements may be expressed, such as “How do I stop getting headaches?”, “How can I get immediate relief from a headache?”, “What is the best painkiller for headaches?”, “The best painkiller for headaches?”. Entities provide a specific context to fulfill these intents. For example, in the utterance “Why am I having headaches at night?”, we can extract two entities, both what kind of symptom that the user notices (headaches) and the time that the symptom occurs (at night). NLU can be either hand-crafted based on domain knowledge, or trained on task-specific labeled data. We focus on the latter, as this is then applied to model the dialogue in an end-to-end manner.

Many related works have been done in developing such techniques in entity classification on EHR data. Liu et al. [33] used LSTM for clinical entity recognition and protected health information recognition. Jagannatha et al. [34] also applied LSTM for classifying relations from clinical notes and extended conditional random fields (CRFs) to improve the accurate phrase detection of various medical entities. Wei et al. [35] presented bidirectional recurrent neural networks (Bi-RNNs) and CRFs for disease and chemical entity recognition in scientific articles. Chlapathy et al. [36] combined Bi-LSTM-CRF for clinical concept extraction. Although these studies showed the positive impact of entity extraction, we need to consider how the extraction model is built upon large and high-quality labeled data, which is expensive to obtain, especially in the nursing care domain that requires domain experts to label them. In contrast, several studies investigated the method of clustering texts to eliminate this need. Wang et al. [37] adopted the dependency-based word embeddings to cluster medical terms (e.g., symptoms, antibiotic medications) on clinical notes from the EHR system. Huang et al. [38] used the k-means algorithm to cluster patients according to medical utilization on emergency department (ED) data. Nicole et al. [39] applied latent cluster analysis (LCA) to cluster among elderly ED patients. Sobhani et al. [40] presented the Non-negative Matrix Factorization (NMF) for argument-tagging based on topic modeling. Kim et al. [41] presented a divisive hierarchical clustering technique to identify clinically interesting sub-populations in EHR data. However, the clusters on their own do not provide any form of utterance. To the best of our knowledge, our study is the first to formalize standard nursing records as dialogue data structures.

### 2.2. Nursing Care Recording

Healthcare is undergoing dramatic changes driven by digital technologies. The transformation from paper to EHR has unquestionably increased the performance and productivity of nurses. It enables quick access to patient records and a share of electronic information with other providers for more efficient care.

A typical EHR interaction is based on graphical user interfaces (GUI) (e.g., input field, select form, checkbox, or radio box), either computers or smartphones [42,43]. However, the typing speed is slow, and the error rate is relatively high for taking long notes, especially those who are inexperienced and unfamiliar with computer screens and keyboards. Nurses may also be unable to record in real-time if their hands are not free, such as the situation where they are taking care of patients. Speech recognition technology allows users to interact with voice only and helps to capture speech at a faster rate than typing [2,3]. Moreover, with the capabilities of the voice assistants and natural language processing (NLP), users do not need to remember an exact command or syntax to control the device with spoken commands. The results of our previous experiment [44] showed that integrating the voice assistant into EHR systems can help to minimize documentation errors and time. Although it is still hard to replace GUI wholly with the voice, it creates the best user experience in many situations, as we mentioned above.

In this work, we focus on EHR for nursing records, especially recording activities of direct care for the elderly, different from general EHR systems that are used for entering information on new patients or updating with each new encounter. In care homes, nurses usually provide patients with nursing care up to 24 h a day, and often perform two or more activities simultaneously. Thus, the record of activities can have many applications, like the execution time of activities and record care routines. The details of activities and records will be described in Section 3.1.

### 2.3. Data Augmentation

Data augmentation is an approach used for increasing the diversity of data available for training models, without actually collecting new data. It is widely used in the field of image transformation in computer visual areas, such as to crop, rotate, or mirror an image without changing the original label. In the NLP task it is much more complicated, as it is challenging to preserve the contextual and grammatical structure of language texts.

A useful method is replacing words or phrases with their synonyms. Zhang et al. used a word from the thesaurus obtained from WordNet, where the geometric distribution [45] ranks every word or phrase synonym. Thesauruses are alternative resources for NLP tasks, which gather words according to similarity. Wang et al. [46] presented k-nearest-neighbor (KNN) and cosine similarity of word embeddings to find a similar word for replacement. Instead of using static word embeddings, authors in [47] used contextualized word embeddings to replace the target word. The author in [48] also proposed a bi-directional language model to predict possible replacement by giving surrounding words. Kafle et al. introduced a generation method for visual question answering by replacing the whole sentence rather than a single one of few words [49]. The authors of [50] presented easy data augmentation techniques by combining four operations, including synonym replacement, random insertion, random swap, and random deletion.

Paraphrasing user utterances is another approach that can be applied to increase training sets to enhance model performance. Barzilay et al. [51] presented an unsupervised approach using multiple-sequence alignment to paraphrase utterances. Kauchak et al. [52] applied the paraphrasing method in the context of machine translation by finding a paraphrase of the reference sentence that is closer in wording to the machine output than the original reference. Quirk et al. [53] applied statistical machine translation (SMT), where translations are generated based on statistical models to generate novel paraphrases of input sentences in the same language. Zhao et al. [54] also used a statistical model to generate paraphrases. Sennrich et al. [55] presented another approach used for machine translation: the back translation technique (i.e., translation of the target language back into the original language). Furthermore, several works have recently been a focus on crowdsourcing to increase the utterance variations from user feedback [56,57]. Among all these, we extend some augmentation approaches to solve our problems. Details are in Section 3 in generation processes.

### 2.4. Feature Extraction

The traditional strategy for representing text data for a machine learning algorithm is a bag-of-words (BOW) model. It represents each text document as a numeric vector by calculating the frequency that each word appears in a document. BOW consists of two conventional approaches: CountVectorizer and Term Frequency Inverse Dense Frequency (TF-IDF) [58]. CountVectorizer converts the text document collection into a matrix of integers, while TF-IDF transforms a count matrix into a normalized TF-IDF representation. BOW offers better performance when positioning or contextual information is not relevant; however, it also has some limitations, such as large feature dimensions and sparse representation.

Word embedding is an alternative technique that convert words or phrases to vectors of real numbers in a low-dimensional space relative to the vocabulary size. Since similar words have similar representation, they therefore it can be used to address the limitation of the BOW representation. The most popular word embedding model is Word2Vec, which learns word embeddings using a shallow neural network. Two more popular methods are the continuous bag-of-words (CBOW) model and the skip-gram model [26]. The CBOW model predicts the current word from a window of surrounding context words, while the skip-gram uses the center word to predict the surrounding words instead. Global Vectors (GloVe) was proposed by [59] and works quite similarly to Word2Vec, but learns vectors from their global co-occurrence information. Its loss function is calculated by taking the difference in the product of word embeddings and the log of the probability of co-occurrence. FastText [27] extends the Word2Vec model but represents each word as an n-gram of characters. Thus, the vector for a word is composed of the sum of these character n-grams.

In recent years, several studies have focused on a contextual embedding technique that captures the context of a word and takes word order into account, rather than outputs with one embedding for each word. Elmo [28] representation is a contextual and character-based model. It uses morphological information to form representations even for out-of-vocabulary (OOV) tokens. Bidirectional encoder representations from transformer (BERT) [30] also incorporates context using a transformer-based model with positional encoding to represent word positions. Instead of solely averaging embeddings of the words, sentence embeddings propose to embed a whole sentence into a vector space. Google presents universal sentence embedding (USE) [29], which is trained on large corpora and results in better generalizable sentence representations for multiple downstream tasks. Another famous sentence encoder model is InferSent [9], which outperforms the results obtained by the SkipThought vectors. It is trained on natural language inference data and also generalizes well to different tasks.

Since there is no clear evidence in previous research that show how one architecture outperforms others, most of the earlier studies are designed to solve problems that authors are facing in their problems. In the following, we examine some approaches that are given above and compare them to each other to find the best setting for our task.

## 3. Dialogue Generation

To train the NLU for accurate intents and entity extractions, it is necessary to capture a variety of different example utterances for each intent. However, there are no publicly available NLU datasets of the nursing care domain. We cannot begin with training a text generation model, such as a typical stacked RNN or sequence-to-sequence (seq2seq) [60], since it needs data to learn sequence pairs to generate one from the others. Furthermore, it is laborious to create and annotate a large number of utterances manually. To address these challenges, we propose to apply data augmentation techniques and the rules of linguistics syntax to generate varied utterance patterns. In the following subsections, we describe the model of care details associated with activity classes and constructing utterances in more detail.

### 3.1. Data Modelling

In contrast to the way users converse with a virtual assistant, the traditional natural language uses considerably complex conversations which tends to be simple commands and directly specific actions. Thus, it can be defined as semi-structured, as it contains more semantic tags or entities. In our prior work on a dialogue-based annotation for activity recognition [61], we suggested that for the usage across all users, there were very general action words and name-specific activities (e.g., show activity, start sitting, stop walking), with 47.7% being just two words and 35.7% being a single word. Thus, we designed our utterances based on action-driven intents and avoided long sentences to accomplish the task efficiently. Each intent consists of a verb that indicates an action and an activity class that supports the action of the verb (e.g., #add-vital, #clean-oral, #prepare-meal, #change-diaper, #assist-toilet, #assist-bath). Note that this concept is related to the “dobj” edges of a dependency tree (direct object of a verb phrase), as shown in Section 4. The data model is flexible, based on the different records of each activity type. Each model mainly includes the following information:

- Nursing activity class (e.g., vital signs measurements, blood collection, diaper exchange);
- Information for one or more target residents, depending on the individual business (e.g., a patient’s name, a patient’s room number);
- The execution time of activity (start and finish times);
- Care records linked with activity (e.g., blood volume for blood collection).

**Figure 2:**
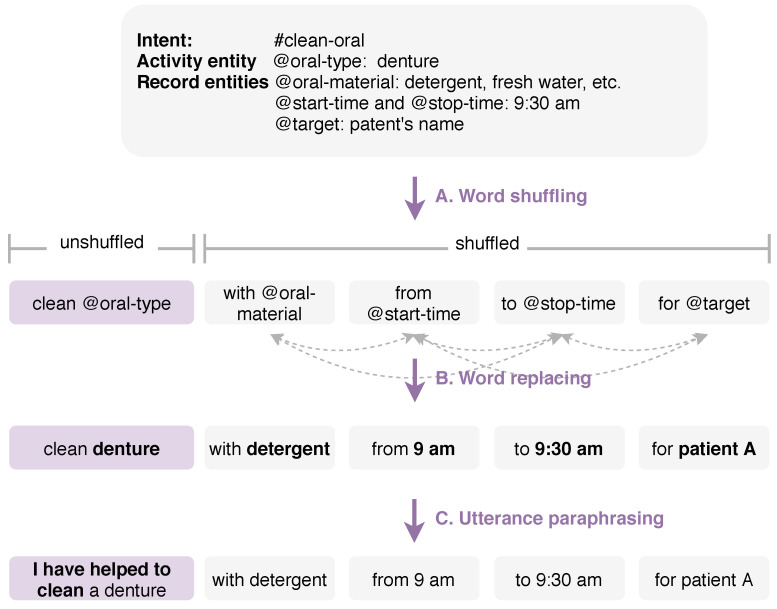
An example of the utterance augmentation task. The task is carried out with the following steps: (A) word shuffling; (B) word replacing; and (C) utterances paraphrasing.

Given is an example of a data model oral assistant activity (#clean-oral), as shown in Figure 2. Nurses desired to record the oral assistance that they provided to patient A within the interval of 9:00 a.m. to 9:30 a.m. The intent #clean-oral can have @oral-type as the activity class entity and @oral-material, @start-time, @stop-time, and @target as the record entities. The @oral-type can have values such as mouth and denture cleaning. The @oral-material can have various fields belonging to @oral-type, such as sponge brush, interdental brush, dental floss for mouth cleaning, or detergent and freshwater for denture cleaning. Different time expressions can represent the @start-time and @stop-time, for example, an exact time (at 10:30 p.m., at half-past six), a period (in the morning), a duration (for two hours), and a point in time (since 11 o’clock). The @target can be the patients’ information, such as the patient’s name, the patient’s room number, and a group of patients.

In this paper, we employed nursing activities and recorded information performed in a real nursing care facility in Japan as an ontology (see Appendix A for details). We selected the activities in which nurses performed more than a five-minute interval. In total, we generated 3546 sample utterances with six intent types, each of which contains different unique entities. An example of intents and entities are presented in Table 1. The statistics of the data are described in Table 2.

### 3.2. Utterance Augmentation

To create initial data for model training, we performed a set of defined data with augmentation operations as the following steps:

#### 3.2.1. Word Shuffling

Figure 2-A shows an example of a word shuffling step. We first created a list of prepositional phrases of record entities to make a complete and coherent phrase in which entities are reduced to placeholders (referred to @). A prepositional phrase is a group of words containing a preposition, a noun, or pronoun object of the preposition, and any object’s modifiers. In our approach, we used two common phrase patterns:

1. Preposition + Noun, Pronoun, Gerund, or Clause
2. Preposition + Modifier(s) + Noun, Pronoun, Gerund, or Clause

(1) A prepositional phrase will begin with a preposition and end with the entity (e.g., on the second floor, before the meeting, at the room, under the bed). (2) The preposition’s object can have one or more modifiers to describe it (e.g., in my room, on his front porch).

Then, we shuffled those entities in the list created if they did not reflect the meaning of the sentence by considering the ordering of subject, object, and verb in a transitive clause (e.g., subject-verb-object order). We created iterators for finding all possible combinations and accessing them one by one. To avoid duplicate entries, we kept a state of the previously generated list and compared it. We skipped it if it had already been used. Subsequently, we connected them with the activity entity to create a new utterance.

#### 3.2.2. Word Replacing

Figure 2-B shows an example of a word replacing step. We replaced placeholders with their real values and expression synonyms. For example, @oral-type was replaced by mouth and denture; @oral-material was replaced by sponge brush, interdental brush, and dental floss; @time was replaced by 8 a.m. and eight o’clock; and @time-range was replaced by the morning, afternoon, and evening.

To find synonyms in words for replacement, we proposed a pre-trained Word2Vec model and cosine similarity. We used the pre-trained Google Word2Vec model [62] that trained on roughly 100 billion words from a Google News dataset. It contained 300-dimensional embeddings for 3 million words and phrases. We loaded the model and looked up the top 10 words that positively contributed to the similarity with a higher count threshold of 0.5 using Gensim [63]. The similarity was determined using the cosine distance between a simple mean of the projection weight vectors of the given words and the vectors for each word in the model. Some abbreviations and units of measurement (e.g., mmHg, bpm, cm, kg) may have included special characters, so these were removed before application.

Some unique words, such as medical and scientific terms, may not exist in the vocabulary of the pre-trained word embeddings model. Instead, we retrieved data from Wikipedia and focused on anchor text (i.e., the tag) in the first paragraph of the article. The anchor text typically links to other related articles. For example, the article on dysphagia contains the text “swallowing”, and the article on swallowing also contains the text “deglutition”.

#### 3.2.3. Utterance Paraphrasing

Figure 2-C shows an example of an utterance paraphrasing step. We reformatted utterances using syntactic transformation techniques that we looked over from the two benchmark NLU datasets, Snips [64] and Kvret [21]. A transformation is defined by the rules whereby words or other elements of sentence structure are combined to form grammatical sentences. Both datasets were collected using different methods, but had a similar syntactic structure to ours.

- Snips: Datasets were collected from real-word usage of chatbot and voice assistant platforms. We chose the Snips dataset performed in June 2017. It contained custom intent engines from 5=five platforms, including Google’s Dialogflow, Wit.ai, Microsoft’s Luis, Amazon’s Alexa, and Snips Voice Platform. This dataset contained 2400 queries for each of seven user intents: add to playlist, play music, book restaurant, get weather, rate book, search creative work, and search screening event.
- Kvret: The dataset using a Wizard-of-Oz scheme which incorporates crowdsourcing (Amazon Mechanical Turk platform) for data collection. Thus, utterances tend to be correct compared to real Snips. The dataset contained intent and entity annotations for 3031 multi-turn dialogues associated with in-car voice assistants. The three different sub-domains are provided, including calendar scheduling, weather information retrieval, and point-of-interest navigation.

From these datasets and their techniques, we inferred and designed the set of transformation following four rules:

- Change statement into question forms with Wh-questions (e.g., what, when, where, who, whom, which, whose, why, and how) and Yes/No questions (e.g., be, do, have, or a modal verb). For example, from “add 80 beats” to “Can you add 80 beats to a patient A?”;
- Insert “I” or “Please” words before the activity entity. For example, “Please, put this 155 cm on the record of a patient A”;
- Transform a sentence from an active to passive voice. For example, transform from “I have cleaned dentures with detergent” to “Dentures are cleaned with detergent”;
- Replace keywords with synonyms, as described in Section 3.2.2. For example, “Clean” is replaced by “wash, scrub, wipe, sponge”, and “Add” is replaced by “attaching, put, set”.

### 3.3. Utterance Generation

Since we started with an augmented-based model, we wanted to supply future models on deep generative models as alternatives to scalable data. Thus, we used a set of utterances in the previous steps to train a character-based LSTM model. To create utterance generation, we built the model using the following steps:

#### 3.3.1. Model Selection

With the rise of advancement in research in NLP, especially in mainly text generation tasks, these approaches use standard recurrent neural networks (RNN). LSTM is an improvement over the general RNN, which possesses a vanishing gradient problem by incorporating gating functions into their state dynamics and utilizes a memory cell that may maintain its state value over a long time. LSTM contains three non-linear gates, namely, an input, an output, and a forget gate. The input gate decides what new information we are going to store in the cell state. The forget gate decides what information we are going to throw away from the cell state. The output gate decides what we are going to output.

We trained a generation language model using the character-based LSTM on our augmented dataset, which will be used to generate new utterances. We arbitrarily chose the character-based one for the preliminary experiment because the fastText one, based on the character n-grams model, had shown the highest accuracy compared to other embedding models for our clustering tasks. The basic idea is that we trained the model to predict the next character in the sequence based on the probability distribution of the last character of the current sequence and repeated sequence of characters for creating words or sentences. For example, given a sequence of characters “vita”, the next character is predicted as “l”, and the result will be “vital”. In the following subsections, we describe the implementation of the generation model step-by-step.

#### 3.3.2. Data Preparation

We split the text into sequences with a fixed length of 100 characters. Each sequence is followed by a target input, which moves one character step from the 100 character input window. Thus, characters of timesteps 0 to 99 in the sequence, the model predicts characters of timesteps 1 to 100. For example, if the sequence length is 4, the input sequence would be “vita”, and the target sequence would be “ital”. To avoid a large vocabulary size, we calculated the frequency for each of the characters. Then, we mapped each character to a unique integer, including white-space and newlines (n). We added n at the end of each utterance to facilitate the model’s capacity of learning how to finish the creation of the new sequence. For example, [add pressuren] characters mapped to int [12 15 15 1 27 29 16 30 30 32 29 16 0]. These numbers of sequence length were fed in at each step of the training.

#### 3.3.3. Generation Model

Since the dataset has just 136,389 characters and only 38 unique characters in the vocabulary for the network to learn, we defined the model with a simple three-layer stack using Tensorflow [65].

**Figure 3:**
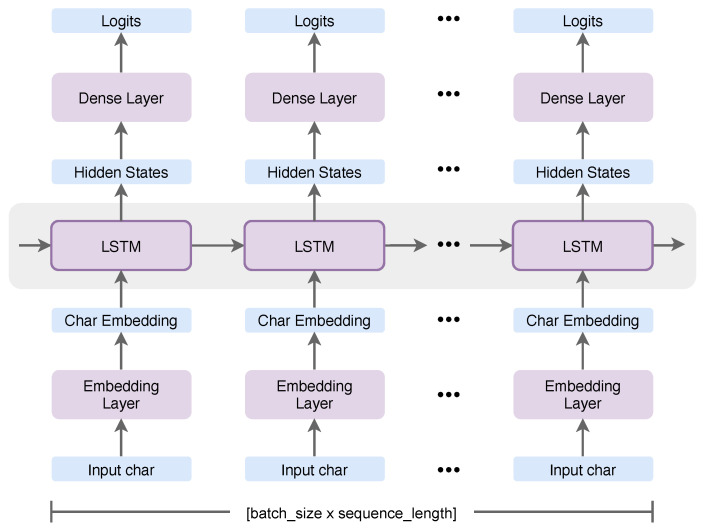
The architecture of a character-based model based on long short-term memory (LSTM). The first layer is a hidden embedding layer. The second layer is a single hidden LSTM layer. The last layer is a dense layer that was applied with the categorical cross-entropy loss function.

Figure 3 reflects a high-level understanding of the model. The first layer is an hidden embedding layer that projects each character into a character lookup table with 256 dimensions. The second layer is a single hidden LSTM layer with 1024 memory units with a probability of 0.2 dropouts that returned the hidden state output for each input time step. The last layer is a dense layer that was applied with the categorical cross-entropy loss (softmax loss) function to output a probability prediction for each character between 0 and 1. The model was trained in mini-batches of 64 over 30 epochs.

After training was finished, we restored the weights of the model and used it to generate new utterances. We fed a character into the RNN and obtained the most probable next-sequence character from a categorical distribution. We set the probability of the softmax as equal to 1.0, which means the probability for the character to be drawn was similar to the probability for the character to be next in the sequence. We sampled the character from this distribution and fed it right back to get the next character and more contexts from the previously predicted characters. Section 6 shows the results obtained from the model.

## 4. Dialogue Labeling

The semantic similarity measure is the ability to determine how close two pieces of text are, both in surface closeness and meaning. The key idea is to represent documents as fixed-length vectors of features (embeddings), and compare documents for their similarity by calculating the distance between these features.

Our approach leverages semantic embeddings as input features to build the clustering model. We experimented with both word-level and sentence-level embedding models. In the rest of this section, we give an overview of selected word-level and sentence-level embedding models. Next, we explain the process of converting the text into respective vectors and computing the clustering similarities.

### 4.1. Embedding Models

In this study, we selected current strong baselines that have been shown as better word embeddings for most general NLP tasks and several state-of-the-art models that have been recently published in the past few years to create our embedding features. The selected models are as follows: word-level embeddings include skip-gram Word2Vec, fastText, and Elmo; sentence-level embeddings include USE, InferSent, BERT, and Elmo fixed mean-pooling. Each model was trained on our generated dataset and we tuned the model to suit our task except the experiment of InferSent, which was trained on fastText pre-trained embeddings due to its characteristics. A brief introduction of these models has already been discussed in Section 2.4. For a full theory behind these models, we recommend reading from the original literature. We built the training data in a compatible form of input, depending on the training model’s goal. Below is a brief description of the implementation of each model.

- **Word2Vec:** We trained both CBOW and skip-gram models, while the results indicate that the skip-gram model outperformed the CBOW model. We thus present only the results of the skip-gram model. The model was implemented with Gensim. The parameters were tuned as follows: The number of features was 32, the minimum word count was 3, the number of threads was 4, the context window size was 6, and the downsampling for frequent words was $1\times 10^{-3}$. The model was run iteratively until the accuracy was saturated; the optimal number result was 50 epochs. We observed that the number of features could affect the accuracy performance, since increasing more features led to worse results. In contrast, the size of the context windows between 4 and 6 gave similar results.
- **fastText:** As the model considered each word as a bag of character n-grams, the word was represented by the sum of the vector representations of its n-grams (i.e., a subword model). For implementation, we first reformatted our dataset to the format of the fastText model. For example, consider the n-gram where n is 3 (trigrams), the character 3-grams of the word “fever” would be [‘<fe’, ‘fev’, ‘eve’, ‘ver’, ‘er>’]. The special symbols < and > at the beginning and end of words are used to distinguish prefixes and suffixes from other character sequences. Then we learned word vectors with the fastText original package [27] on this data. We also used the skip-gram model and tuned the other parameters with the same values in the Word2Vec model.
- **Elmo:** Generally, the input to the model is a sequence of words, and the output is a sequence of vectors that allows us to perform different tasks based on output. Thus, we exploited the model to build both word and sentence embeddings. We used TensorFlow Hub (TF-Hub) (https://www.tensorflow.org/hub) to load the Elmo pre-trained model to then pass a bunch of text inputs to the model. The model outputs fixed embeddings at each LSTM layer. We used the weighted sum of the three layers with word embeddings for word-level features, and a fixed mean-pooling of all contextualized word representations for sentence-level features. Each word and sentence was a vector size of 1024.
- **USE:** We used TF-Hub to load the pre-trained USE model that was trained with a Transformer. We then used this model to create embeddings for our sentences. The models were trained on a variety of sources, such as Wikipedia, web news, discussion forums, and supervised data from the Stanford Natural Language Inference (SNLI) corpus. The model returned vectors of 512 dimensions as output, irrespective of the length of the input. Each sentence had a vector size of 512 with normalized values.
- **InferSent:** InferSent used a supervised learning approach to generate sentence embeddings, which need to have labeled data in advance of running the algorithm. We used the InferSent, which trained with the pre-trained fastText model instead and updated only the vocabulary of word vectors with our dataset. The model was adopted by a bi-directional LSTM with a max-pooling operator as a sentence encoder and trained on the SNLI corpus. The output of the model encoded sentences in fixed-length vectors of dimension 4096.
- **BERT:** We used TF-Hub to load a pre-trained bert-base-uncased to build sentence embeddings. The model was trained on lower-cased English, which has 12 layers and 768 hidden states. Unlike USE and InferSent, which can directly consume a list of sentences, BERT requires the inputs to be pre-processed. Thus, we used the BERT tokenizer to tokenize sentences into smaller subwords and characters. This kind of tokenization will help to deal with OOV words and complicated words. We also added a special [CLS] token at the first position, and [SEP] at the end of the sentence for separating sentences for the next sentence prediction task. For example, the output of a pre-processed sequence is [‘[CLS]’, ‘add’, ‘pressure’, ‘with’, ‘103’, ‘s’, ‘##ys’, ‘##to’, ‘##lic’, ‘blood’, ‘pressure’, ‘[SEP]’]. Each sequence is truncated down to 20 as the maximum sequence length. Then, we took the tokens input and passed it to the BERT model. Each sentence was a vector size of 768.

### 4.2. Word-Level Features

In this section, we create word features following these methods. In order to facilitate our experiments, a few normalization steps had to be performed on our dataset to reduce the feature space. We first expanded word contractions (e.g., “I’m” is replaced with “I am“) and produced as lower case. We then tokenized text into a list of tokens using spaCy (https://spacy.io). For example, the output of tokenization is [‘add’, ‘pressure’, ‘with’, ‘103’, ‘systolic’, ‘blood’, ‘pressure’]. For efficiency, we limited retrieved results to tokens with a minimum length of four characters and ignored stopwords (e.g., a, an, the). We then normalized the word with lemmatization that gets synsets from WordNet (e.g., closest replaced with close) and removed non-ASCII letters, including punctuation, spaces, and special characters. Its implementation relies on regular expressions.

We created sentence embedding (except for Elmo) by using the averaging approach for aggregating the word embeddings since it consistently gives reliable results. Given an utterance $U=\{w_{1},w_{2},\ldots ,w_{N}\}$, we transformed each word into a vector representation $v_{w_{n}}$. Subsequently, we averaged out all the word vectors of the utterance as the word-level feature:

$$F_{w}=\frac{1}{N}\sum_{n=1}^{N}v_{w_{n}}$$

### 4.3. Sentence-Level Features

At the sentence-level, we only removed non-ASCII characters. Since lemmatization aims to reduce inflectional forms of a word to a common base form, which may cut off some semantic contexts, for example, “close”, “closer”, and “closest” have the same root word (in an adjective form), but their semantic meaning is different from each other. Each sentence is represented as a fixed-length vector representation. Given an utterance $U=\{w_{1},w_{2},\ldots ,w_{N}\}$, the utterance is represented with embeddings using each pre-trained model:

$$F_{s}=\left\{s_{1},s_{2},\ldots ,s_{n}\right\}$$

### 4.4. Intent and Entity Labeling

Since entities are smaller parts (words) of the intent (sentence), we used only word-level embeddings for entity clustering, while we used both word-level and sentence-level embeddings for intent clustering.

As most of the entities were composed of noun phrases (i.e., a noun plus the modifiers which distinguish it), we split the utterance into noun phrases using dependency parsing in spaCy, then mapped them with their corresponding ground truth. Dependency parsing facilitates this process by identifying the relationships between noun phrases in the utterance. It transforms a sentence into a dependency tree, a structure that can be defined as a directed graph, with vertices corresponding to the words and arcs corresponding to the syntactic dependencies between them. These relations give details about the dependency tag (e.g., nsubj: nominal subject, det: determiner, nummod: numeric modifier, obj: object).

**Figure 4:**
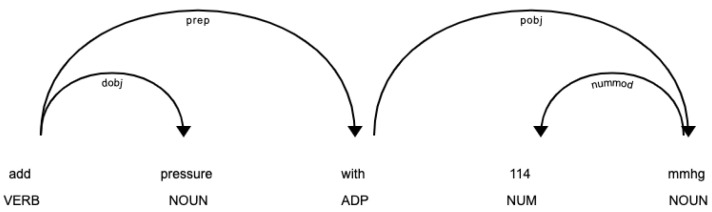
An example of a dependency parse of a sentence that represents its grammatical structure and dependencies.

For example, regarding the utterance in Figure 4, “add pressure with 114 mmhg”, it consists of two nouns: “pressure” that describes the vital, while “mmHg” describes the unit of pressure. The dependency tag under the arcs denotes a prepositional modifier, which modifies the meaning of the noun. For instance, “pressure” is linked to the root “add” as the object of the verb (dobj); “mmHg” is linked to the root “114” as a numeric modifier of a noun (nummod) to modify the meaning of the noun with a quantity, and “114 mmHg” is linked to the root “with” as the object of a preposition (pobj).

### 4.5. Clustering Model

The task here is given a list of utterances. We clustered them so that semantically similar utterances were in the same cluster. According to this procedure, we utilized k-means clustering [66], one of the most widely used clustering algorithms. The algorithm iteratively moves the k-centers and selects the data points closest to that centroid in the cluster. This method is defined by the objective function, which tries to minimize the sum of all squared distances within a cluster for all clusters. The objective function is defined as:

$$J=\sum_{j=1}^{k}\sum_{i=1}^{n}{∥x_{i}^{\left(j\right)}+c_{j}∥}^{2},$$

where *k* is the number of clusters, *n* is the number of cases, $x_{i}$ is a case *i*, and $c_{j}$ is a centroid for cluster *j*. Since the k-means algorithm needs a random initialization, we used k-means++ to choose initial cluster centroids. The number of clusters *k* is set to be equal to the number of classes the dataset generated (i.e., the gold standard *k*). We trained clustering for each feature embedding. Each cluster was assigned to the class, which was the most frequent output label among the members in this cluster, and computed the average accuracy of such assignments’ overall clusters.

## 5. Experimental Setup

In this section, we described the experimental setup and evaluation metrics used for the generation and the labeling evaluations.

### 5.1. Dialogue Generation Evaluation

We evaluated both the dataset was generated from the augmentation technique, and the dataset was generated from the LSTM model.

#### 5.1.1. Augmentation Evaluation

The augmented dataset was evaluated using human judgment, which focused on two hypotheses:

- Fluency: How well does the utterance perform in both being more natural to read and comprehend?
- Accuracy: How well does the utterance perform in both its grammatical correctness and adequacy?

To explore these, we experimented with two different participant groups to perform a questionnaire. First, we employed domain expertise related to the nursing care domain. We requested three people working at Chulabhorn hospital (https://www.chulabhornhospital.com) as a nurse and nursing assistant to rate 100 random utterances from the dataset. Second, we crowdsourced 300 diverse workers on an Amazon Mechanical Turk (MTurk) (https://www.mturk.com) to perform. We narrowed down the workers using MTurk qualifications to help us target suitable workers for our tasks. Workers were filtered based on their literacy level of English language (native speakers or non-native speakers who can speak English fluently) and their prior experience with the voice assistant technology. We randomized 50 utterance samples for each intent from our dataset and accumulated human ratings from independent workers for each output. In total, we gathered 300 responses from them.

The questionnaire was designed to request each participant to specify his/her level of satisfaction to the utterance on a 5-point Likert scale: (5) strongly agree, (4) agree, (3) neutral, (2) disagree, and (1) strongly disagree.

#### 5.1.2. Generation Evaluation

We generated 1000 samples from our LSTM trained model and examined all utterances that were generated manually. We inferred each generated utterance from its ground-truth label, and if it consisted of multiple intents, we would leave them out. In total, we agreed on 86.4% of meaningful utterances.

We then evaluated the similarity between the generated utterance and its reference utterance using the Bilingual Evaluation Understudy (BLEU) [67] and averaged all the results. BLEU is a metric for evaluating a generated sentence to a reference sentence using the concept of modified n-gram precision and brevity penalty. The author in [68] showed that these metrics could show a comparatively stronger correlation with a human assessment on task-oriented datasets. A perfect match results in a rating of 1.0, whereas an absolute mismatch results in a score of 0.0.

### 5.2. Dialogue Labeling Evaluation

To evaluate intent and entity labeling, we used two different metrics for automatically measuring the quality of the produced clusters, f1-score, and silhouette coefficient, defined as follows:

#### 5.2.1. F1-Score

F1-score used to compare the results of each algorithm against its ground truth for quantifying the quality of predictions. It presents the balance between precision (P) and recall (R) and reaches its best value at 1 and the worst score at 0. To apply the f1-score to the precision and recall of pairs, we defined pairs of items in each cluster:

- True positive (TP): the number of item pairs in the same cluster and which belong to the same class;
- False positive (FP): the number of item pairs in the same cluster but which belong to different classes;
- True negative (TN): the number of item pairs in different clusters and which belong to different classes;
- False negative (FN): the number of item pairs in different clusters but which belong to the same class.

Then, we computed P, R, and the F1-score as follows:

$$P=\frac{\mathrm{TP}}{\mathrm{TP}+\mathrm{FP}}\;\;R=\frac{\mathrm{TP}}{\mathrm{TP}+\mathrm{FN}}\;\;F1=\frac{2\mathrm{PR}}{P+R}$$

#### 5.2.2. The Silhouette Coefficient

The silhouette was used to evaluate clustering results. Intuitively, it computes how similar a point is to its cluster (cohesion) compared to other clusters (separation). The silhouette coefficient is calculated as follows:

$$S\left(i\right)=\frac{b(i)-a(i)}{max(a(i),b(i))},$$

whereby *i* represents the data point, a(i) is the mean intra-cluster distance, and b(i) is the mean nearest-cluster distance for a data point. From this definition, we can see that the silhouette width s(i) is always between $\left[-1,1\right]$. A high value indicates that points are well-matched within clusters and poorly between clusters, whereas a low value corresponds to the opposite. Therefore, we assumed that we could achieve the highest values s(i) at the goal standard *k*.

## 6. Results

In this section, we present our empirical results. We start by describing the dialogue generation experiments and then detailing different models applied for dialogue labeling, along with their results.

### 6.1. Dialogue Generation Results

We named the dataset was generated from the augmentation method as ‘augmented dataset’, and the dataset was generated from deep-learning models as ‘generated dataset’. Utterance samples in the dataset are shown in Table 3.

#### 6.1.1. Augmented Data Quality

Table 4 shows crowdsourcing evaluation results. Overall, we found the dataset to be generally acceptable by participants in both groups (4.71 ± 0.58 ratings for fluency scores and 4.66 ± 0.57 accuracy scores). The scores from participants with high levels of domain expertise performed a little better than crowd workers (+0.4 for fluency scores and +0.6 accuracy scores). We directly asked the participants in the first group to give opinions on low score utterances. They provided a similar main reason, that is, that some words were difficult to understand because they had never seen them before. We found that these words were synonym words generated by the pre-trained Word2Vec model. Thus, the similar words should be carefully developed and reviewed by subject experts. Conversely, we found that utterances of #add-vital intent were most often given low ratings in the second group; this might have been because the hard medical definition of vital signs was not clear to them.

#### 6.1.2. Generated Data Quality

**Figure 5:**
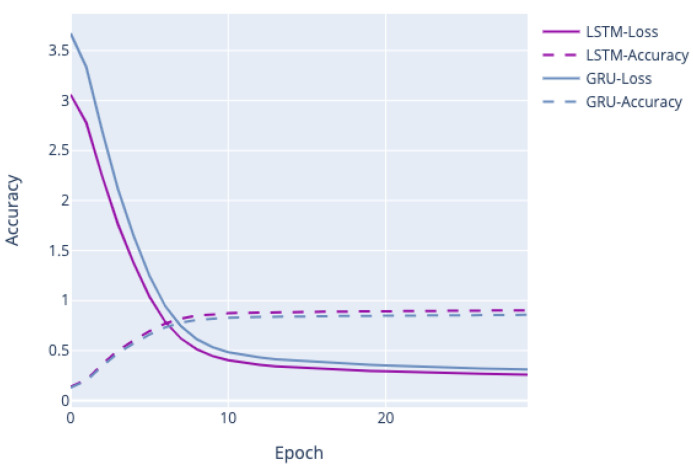
The performance of generation models.

We also performed experiments to see how the performance of generation models was trained in our data schema. Here, we conducted experiments on the following two models. The first model is LSTM, as described in Section 3, and another is a Gated recurrent unit (GRU) [69] as its related LSTM. (LSTM has three gates, namely, input, output, and forget gates, whereas GRU has just two gates, namely, reset and update gates). All parameters were set to the same value in both models. Figure 5 shows the performance of both models. We can see that accuracy and loss have not converged. LSTM achieved 90.28% accuracy and GRU 89.61% accuracy. Since LSTM was slightly better than GRU by 0.67%, we will only discuss the results obtained from the LSTM model.

The results of our generated utterances closely resembled the original dataset (0.76 BLEU scores). In general, generated utterances began with the word or phrase related to the original dataset. Although some utterances were grammatically incorrect but semantically acceptable, this was probably due to how they were trained on the character-based model. As we can see in Table 3, most spelling mistakes are just one or two characters wrong. However, the output, in this case, has less diversity. As we tried to increase the probability of sampling a class (sampled softmax) for more random predictions, the entire new text could give more spelling mistakes and almost complete nonsense. We believe that increasing training samples may help in producing more surprising utterances.

### 6.2. Dialogue Labeling Results

We performed experiments on the embedding models, as mentioned in Section 4. We also reported the results obtained from these models in this subsection.

#### 6.2.1. Comparison Embedding Models

The experimental results are shown in Table 5. For sentence-level representation, USE outperforms all other embedding approaches. USE results in an accuracy of 0.775 f1-scores for intent clustering and 0.585 silhouette scores for entity clustering. For word-level representation, there might be no significant difference between the accuracies of fastText and Word2Vec for both intent and entities as both were trained using the skip-gram model. However, fastText performed slightly better than Word2Vec. fastText improved the f1-scores for intent clustering from 0.795 to 0.798 (±0.003) and for entity clustering from 0.741 to 0.787 (±0.046). Thus, fastText improved the silhouette scores for intent clustering from 0.648 to 0.674 (±0.026) for entity clustering from 0.603 to 0.614 (±0.0.011).

Comparing between word-level and sentence-level, we can see that the overall performance of the vectors generated from word-level embeddings performed better than sentence-level embeddings even when applied for intent clustering. The average of the word embeddings of content words in the utterance of the fastText model shows f1-scores, and the silhouette scores increased on the intent clustering by ±0.023 and ±0.063, respectively. We reasoned that the representations learned from the fastText model included character-based and subword information, which can play an essential role in improving the representations for uncommon words and even OOV words.

Another interesting observation is that all models which show accuracy in entity clustering are worse than intent clustering. In contrast, Elmo performance in entity clustering was better than intent clustering. We suggest that although technically, Elmo is considered a state-of-the-art model and usually yields satisfactory results, they tend to perform poorly on training new embeddings from specialized domains, which may probably be too small. Additionally, its embeddings are contextually dependent, meaning that the word vector changes depending on the sentence it appears in, which sometimes makes them unable to capture semantic meaning in the utterances.

#### 6.2.2. Further Discussion

**Figure 6:**
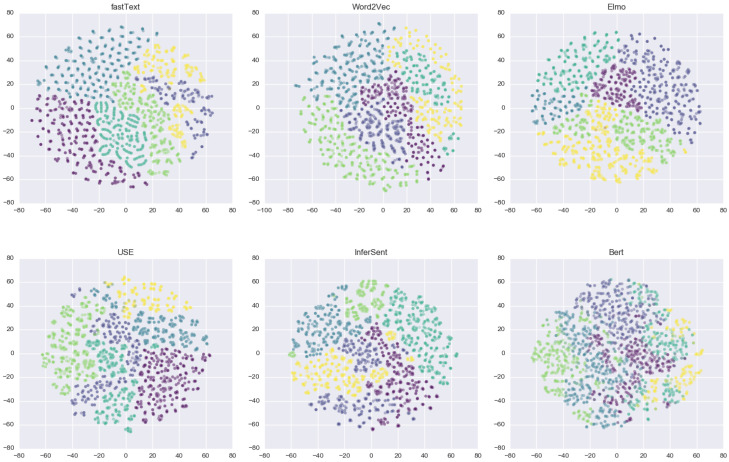
The visualization of sentence clusters trained from six embedding models using t-SNE. Each subplot shows the distribution of sentences within each cluster.

We visualized sentence embeddings in a two-dimensional plot using t-SNE [70] (t-Distributed Stochastic Neighbor Embedding). It is an algorithm for visualizing high-dimensional data, which uses local relationships between points to create a low-dimensional mapping that captures non-linear structures. Example clusters of intents are shown in Figure 6. Each spatial spot in the scatter plot represents the sentence inside a single intent, and similar sentence vectors would be placed in spatial proximity. The color of each spot represents the cluster to which it belongs (e.g., all sentences in cluster #add-vital are represented in yellow, in cluster #assist-toilet are represented in green and so on).

On close observation, it is seen that similar documents (either word vectors or sentence vectors) are occupying adjacent spaces, while different document vectors are scattered in the plot. The model can quickly identify intents and assign them to the cluster if they show apparent dissimilarity between other documents in embedding space, such as #add-vital and #assist-toilet. However, with #assist-toilet and #assist-bath, these items can be difficult to distinguish correctly, as they more closely embed words that occur in the same context. For example, looking at the fastText model in Figure 6. we can see that there are two clusters (yellow and purple), in the general vicinity of each other and almost overlapping, these are clusters of #assist-toilet and #assist-bath.

**Figure 7:**
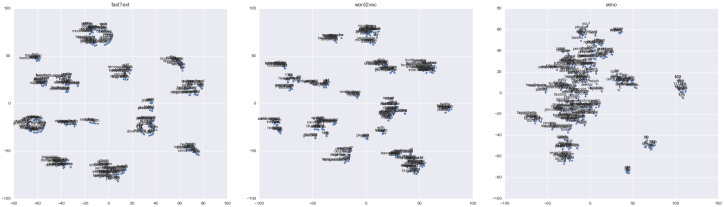
The visualization of word clusters using t-SNE.

Figure 7 illustrates clusters of entities from the fastText, Word2Vec, and Elmo embeddings, respectively. Some words can be one or more entities. For example, the toilet can interpret both places and types in the #assist-toilet intent. One of the most challenging aspects of it is how when the sentence is divided into smaller parts (e.g., word, chunk), it cannot learn the context of relationships within the sentence like we can do in intent clustering. Thus, it is clearly shown that without context rules, it would be challenging to use these words as representations.

Furthermore, as in the generating process, we replaced these target words with their relevant semantic representations using the pre-trained Word2Vec model, where words with a higher probability than 0.5 were used. Although clustering models can easily find similarities and cluster together, some utterances do not make sense, as mentioned in the crowdsourcing evaluation. This problem mainly arises because of words that are synonyms, but they may provide different perspectives in the context of statements. Moreover, since no pre-trained models trained on nursing care records or any EHR data were provided, we used the pre-trained Word2Vec model trained on Google news data, which might have introduced error to obtain word embeddings in the nursing domain.

## 7. Conclusions and Future Work

One of the first steps to automate the construction of task-oriented dialogue systems is automating dialogue labeling to identify the user intent and its adjunct entities in NLU tasks. However, no open data are available, and getting full access to EHR or nursing records is very challenging (e.g., privacy problems). In this paper, we proposed smart ways to produce trained labeled utterances that encompass the functionality required to record information about nursing activities, and also introduced semantic similarity-based clustering using feature embeddings for automatic dialogue labeling. We desired to improve and expand this dataset to make the availability of data that enables better systems to be developed and to share the data with other researchers within the field of training dialogue models to do meaningful research.

We started with creating initial sample utterances using text augmentation techniques. The results show our utterances have a powerfully good impression in terms of both fluency and accuracy scores. We also built a character-based LSTM model to evaluate and understand the opportunities and challenges of using the text generation model trained with our data schema. Although our initial model does not have complex structures and was trained on quite a small dataset, the generated utterances still seem reasonable. In future work, we will look to other recent text generation models that are worth mentioning beyond simple character-based models, such as a bi-directional RNN and generative adversarial network (GAN). Furthermore, we want to increase the number of training data and make utterances to have lots of complexity and multiple variations to ensure that those models do not propagate possible biases present in the dataset.

We experimented with different types of word and sentence embeddings for the labeling problem, intending to gain insights on the embeddings that are most suitable to use with our dataset. We initially began to experiment with six widely used embedding models, including Word2Vec, fastText, USE, BERT, InferSent, and Elmo. Here are a few more variants that we have been trying, with no great success yet. We want to learn other embedding models for further improvement. We observed that fastText outperformed other embeddings on our dataset, while Elmo showed impressive performance for entity clustering. The result shows a type of transfer learning where these pre-trained models can be taken as a base and some modification can work well. We are currently exploring the use of context-based representation techniques for obtaining word embeddings and proposing several modifications to the model. Thus, we will find a possibility of retraining pre-trained word embedding features with our data to increase training data and enhance representation capability.

## Figures and Tables

**Table 1:** Examples of nursing care data include intent, entity, sample values, and sample utterances.

Intent	Entity	Sample Values	Sample Utterances
add-vital	vital-type	blood pressure, body temperature, pulse beats	Can you add pressure with 103 systolic blood pressure?
	vital-value	mmHg, systolic BP, c, celcius, bpm, heartrate	Set temperatures with 39 deg
clean-oral	oral-type	mouth, dentures, partial denture, orthodontic braces	Clean mouth ventilations with fluoridated toothpaste
	oral-material	interdental brushes, dental floss, detergent, water	Wash partial dentures with detergents
ine assist-toilet	toilet-type	portable toilets, port potty, urinals, waterless urinal	I have helped to use urinals on sofa
	toilet-place	toilets, lavatory, restroom, bathroom, loo	help to use porta potty at restroom
prepare-meal	meal-type	breakfasts, lunch, brunch, dinner, meal, supper	Prepare chicken noodle soup for breakfasts
	food-type	noodles, tofu, vegetable soup, chicken soup, fruits	Make udon for supper
assist-bath	bath-type	baths, shower, wipe	Help to baths with bar stool
	bath-material	lift, steal, bar stool, worktable, swivel chair	I have helped to shower with bath transfer chairs
change-diaper	toilet-place	toilets, lavatory, restroom, bathroom, loo	Change dirty diaper at urinal
			Help to change nappies at restroom

**Table 2:** Statistics of the data.

	No. of Utterances	No. of Words	Avg. Utterance Length
add-vital	876	6016	6.867
clean-oral	606	4163	6.869
assist-toilet	408	3074	7.534
prepare-meal	756	4830	6.388
assist-bath	560	3572	6.378
change-diaper	340	2658	7.817
Total	3546	24,313	6.856

**Table 3:** An example of augmented and generated datasets.

Augmented Dataset	Generated Dataset
Add pressure with 103 systolic blood pressure	I help to presture with 88 diastolic pressure
Clean dentures with dishwashing liquid	Please add heartbeat lastorats
Help to use portable toilets at bathroom	Clean porcelain crowns with dish detergent
Help to change soiled diaper at toilet	Help to toliet on foldout couch
Prepare noodles for breakfasts	I have helped to change feeding burping on couch
Help to baths with bar stool	I have helped to change soiled diaperoon slppers

**Table 4:** Crowdsourcing evaluation results. Note: years experience of group 1 is the work experience in hospital, and group 2 is the experience in the use of virtual assistants; M = male; F = female; m = mean; std = standard deviation.

	Group 1	Group 2	Overall
Gender (M,F)	0,3	173,127	173,130
Age (m ± std)	25.6 ± 0.47	36.88 ± 7.98	36.76 ± 8.01
Years experience (m ± std)	1.33 ± 0.47	1.51 ± 1.12	1.51 ± 1.11
Fluency (m ± std)	4.83 ± 0.45	4.59 ± 0.72	**4.71 ± 0.58**
Accuracy (m ± std)	4.79 ± 0.46	4.53 ± 0.68	**4.66 ± 0.57**

**Table 5:** Clustering performance on embedding models. Comparison of word-level representations with sentence-level representations.

Model	Intent		Entity	
	F1-Score	Silhouette	F1-Score	Silhouette
Word2Vec	0.795	0.648	0.741	0.603
fastText	**0.798**	**0.674**	**0.787**	**0.614**
Elmo	0.712	0.405	0.723	0.538
USE	0.775	0.585	-	-
InferSent	0.715	0.421	-	-
BERT	0.667	0.452	-	-

## Appendix A

**Table A1:** List of records.

Activity	Record	Type	Possible Values
Measuring	Maximum blood pressure	Input	greater than or equal to 0
vital signs	Minimum blood pressure	Input	greater than or equal to 0
	Pulse beat (bpm)	Input	greater than or equal to 0
	Body temperature (c)	Input	greater than or equal to 0
	Weight (kg)	Input	greater than or equal to 0
	Height (cm)	Input	greater than or equal to 0
	Note	Text	e.g., being sick, skin colors, have a fever, pain complaint
Meal and	Meal assistance	Select	self-reliance, setting only, partial care, full care
medication	Dietary volume	Select	0 to 10
	Meal size	Select	0 to 10
	Amount of water	Select	0 to 500
	Medication	Select	self-reliance, assistance, no medication
	Note	Text	e.g., dysphagia, appetite loss, using ThickenUp clear
Oral care	Oral cleaning	Select	self-reliance, setting only, partial care, full care, no cleaning
	Denture cleaning	Select	use of detergent, wash in water, no cleaning
	Note	Text	e.g., using sponge brush, using interdental brush,
			using dental floss, oral wound
Excretion	Method of excretion	Select	toilet, portable toilet, urinal, on the bed
	Excretion assistance	Select	self-reliance, setting only, partial care, full care
	Mode of Excretion	Select	defecation, urination, fecal incontinence,
			urinary incontinence, no excretion
	Urine volume	Select	small, medium, large, no choice
	Defecation volume	Select	small, medium, large, no choice
	Type of Waste	Select	watery mail, muddy stool, ordinary,
			hard stool, colo flight, no choice
	Diapering	Select	putt exchange, rehapan replacement,
			diaper change, wipe, vulva cleaning, change assistance
	Note	Text	e.g., hematuria, bloody stools, a tight stomach
Bathing	Bathing method	Select	general bath, shower bath, machine bath, wipe,
			it was planned to bathe but there was no conduct
	Bathing assistance	Select	self-reliance, setting only, partial care, full care
	Use of bath aids	Text	e.g. shower carry use
